# Risk factors for the development of necrotizing enterocolitis in neonatal hemolytic disease: a mini review

**DOI:** 10.3389/fped.2025.1639423

**Published:** 2025-07-29

**Authors:** Haiyue Yu, Wanxu Guo, Yuan Wang, Yunfeng Zhang

**Affiliations:** Department of Neonatology, the Second Hospital of Jilin University, Changchun, Jilin, China

**Keywords:** hemolytic, newborn, enterocolitis, necrotizing, risk factor, intravenous, immunoglobulins, exchange transfusion hemolytic

## Abstract

Hemolytic disease of the newborn (HDN) is a common disease of neonates, and necrotizing enterocolitis (NEC) is a serious gastrointestinal disorder and both occur in the neonatal period that can significantly increase the risk of death in children. Cases of NEC associated with HDN are increasing. The purpose of this paper is to collect and present relevant research data to understand the risk factors associated with necrotizing enterocolitis in neonatal hemolytic disease, and to provide reasonable guidance and reference for clinical work.

## 1 Introduction

Hemolytic disease of newborn (HDN) refers to an alloimmune hemolytic disorder in fetuses or newborns caused by incompatibility between the blood types of the mother and the fetus, with Rh and ABO incompatibility being the most common causes (referred to as Rh-HDN and ABO-HDN, respectively) (1). ABO-HDN has an incidence of approximately 20% (1) and primarily affects term and late preterm infants (2). Rh-HDN, particularly RhD incompatibility, has seen a significant decline in incidence—from around 16% to about 2%—due to the widespread use of antenatal and postnatal anti-D immunoglobulin prophylaxis (3). Nevertheless, Rh-HDN remains a major cause of severe fetal hydrops, preterm birth, and neonatal hyperbilirubinemia.

Necrotizing Enterocolitis (NEC) is a life-threatening gastrointestinal emergency that predominantly affects neonates, especially preterm infants (4). The incidence and mortality of NEC are inversely associated with gestational age and birth weight. In neonatal intensive care units (NICUs), the overall incidence ranges from 2% to 5%, and it rises sharply to 4.5%–8.7% in very low birth weight (VLBW,<1,500 g) infants, with a mortality rate of 20%–30%. Among extremely low birth weight (ELBW, <1,000 g) infants, mortality escalates to 30%–50.9% (5). While histopathological confirmation remains the gold standard for NEC diagnosis, the modified Bell staging criteria serve as a key clinical tool for guiding management and treatment decisions in suspected cases lacking pathological evidence (6).

Notably, recent clinical observations and studies suggest that infants with HDN may face an increased risk of developing NEC during disease progression or treatment (7). The co-occurrence of HDN and NEC not only significantly complicates disease management, prolongs hospitalization, and escalates healthcare costs but also markedly elevates mortality risk. Given these clinical findings and their severe implications, it is crucial to identify risk factors for NEC in HDN patients.

Therefore, this mini-review aims to evaluate existing evidence, focusing on summarizing risk factors associated with NEC development in HDN patients—including intrinsic disease-related factors (e.g., hemolytic anemia, hypercoagulability) and extrinsic treatment-related factors (e.g., intravenous immunoglobulin [IVIG] therapy, blood transfusions, phototherapy), to assist clinicians in early identification of high-risk infants and optimize management strategies, and ultimately improve outcomes in this vulnerable population.

## 2 Risk factors for necrotizing enterocolitis

### 2.1 Intrinsic risk factors

#### 2.1.1 Anemia

HDN is an immune hemolytic disease in which the disease process itself is accompanied by destruction of red blood cells, and the degree of anemia is positively correlated with the severity of hemolysis. Anemia can lead to insufficient perfusion of intestinal blood flow and reduce oxygen supply in neonates, thus causing intestinal mucosal damage (8). At the same time, anemia can disrupt the self-regulatory function of the intestinal vasculature and increase the production of the pro-inflammatory factor interferon-*γ*, leading to further damage to the intestinal barrier (9).

In a prospective, multicenter observational cohort study, severe anemia (defined as a hemoglobin level of 8 g/dl or less) was found to be significantly associated with an increased risk of NEC (adjusted cause-specific risk ratio 5.99 [95% CI: 2.00–18.0]; *P* = 0.001) (10). The case-control study by Jiang et al. (11) suggested that anemia was a risk factor for the occurrence of NEC in neonates without sepsis (Neonatal anemia is defined as Hb levels less than the fifth percentile with Hb levels varying with gestational age). A study conducted by Wang et al. (12) identified anemia as a high-risk factor for the development of NEC in neonates with HDN [OR and 95% CI: 3.568 (1.802–7.065)].

#### 2.1.2 High coagulation state

In HDN, due to erythrocyte rupture, a large amount of erythropoietin and phospholipid coagulase analogs are released (1), which can lead to the induction of potent procoagulant factors in monocytes and endothelial cells, and subsequent coagulation activation. In addition, damaged erythrocytes, activated platelets, and small cell-derived vesicles known as microparticles may promote coagulation by providing a membrane surface containing exposed anionic phospholipids. Some microparticles also contain tissue factor, which further promotes coagulation (13). These results show that the neonates with HDN are often in a hypercoagulable state (14, 15), which may cause small intestinal thrombosis, and in turn leads to intestinal ischemia, necrotic detachment of the intestinal mucosa, and impairment of the intestinal barrier, ultimately leading to NEC. In a retrospective study of 275 full-term newborns with ABO hemolytic disease, the findings show that the development of NEC in neonates with HDN is associated with their degree of endogenous coagulation and platelet adhesion (16). It is considered that the hypercoagulable state of HDN neonates is one of the risk factors for the development of NEC, but further studies are still needed to verify this statement.

### 2.2 Extrinsic risk factors

#### 2.2.1 Phototherapy

Phototherapy (PT) produces isomers of bilirubin by transforming bilirubin so that bilirubin changes from fat-soluble to water-soluble and is excreted from the body via bile or urine without binding by the liver (1). Phototherapy is a simple, noninvasive, and effective method of removing plasma unconjugated bilirubin, which reduces the need for blood exchange therapy and is the priority of treatment for neonates with HDN (1).

Phototherapy is usually considered safe and harmless, but in recent years the potential risks of phototherapy have surfaced with intensive research and the decreasing gestational age and weight of viable preterm infants. Sivakumar et al. (17) found that end-diastolic blood flow velocity in the superior mesenteric artery was accelerated after phototherapy, suggesting that diastolic alterations in mesenteric vasculature may have occurred during phototherapy, resulting in mesenteric ischemia, which caused the patient's intestinal hemodynamic changes. Phototherapy causes difficult closure (18) or even reopening (19) of the cardiac arterial catheter in patients, and therefore may lead to subsequent changes in mesenteric blood flow and associated mesenteric ischemia. The patients are prone to dehydration during phototherapy (20, 21), which can lead to blood concentration and result in ischemic changes in the intestine. In addition, phototherapy may cause changes in the intestinal flora (22, 23), electrolyte disturbances (24), and even exacerbated hemolysis (25), all of which may result in further intestinal damage, leading to NEC.

In a recently published retrospective case-control study that included a total of 196 (of which 122 were controls) neonates over a 7-year period, the investigators found for the first time that the incidence of NEC increased with the duration and number of phototherapy (trend *P* = 0.010 and 0.033) and became higher after adjusting for confounders. Multifactorial analysis showed that phototherapy exposure >120 h and >4 times exposure to PT courses were significantly associated with NEC (26), which also confirms phototherapy as a significant risk factor for NEC in neonates with HDN. It was found that during the application of cold light sources for phototherapy in which chest shielding for neonates was used during phototherapy, the timely supplementation of electrolytes and water, and the strengthening of monitoring for neonates with long and frequent phototherapy sessions are conducive to reducing the occurrence of NEC (27).

#### 2.2.2 Intravenous immunoglobulin G

Intravenous immunoglobulin G (IVIG) is a preparation for intravenous infusion made from normal human plasma extracted by low-temperature ethanol and then subjected to a series of purification treatments, and it contains a variety of specific human IgG antibodies. The treatment of HDN with IVIG is to neutralize the anti-erythrocyte membrane antibodies by specific IgG, reduce their production and accelerating metabolism, preventing complement binding, preventing phagocytosis of sensitized erythrocytes by phagocytes, and reducing the erythrolytic effect (28). A large number of literatures confirm that the frequency of blood exchange due to HDN can be significantly reduced after the application of IVIG (29), which is now widely used in the clinical treatment of HDN.

However, NEC occurrence in neonates treated with IVIG continues to be reported in case reports and clinical studies, and as early as 1990, a case report of NEC in a 38-week-old boy with no other risk factors, 3 days after high-dose IVIG treatment, he developed thrombosis due to the high viscosity of the IVIg solution (30). In another controlled trial using IVIG to reduce nosocomial infections in very low birth weight infants, the NEC rate was 12.0% in the IVIG group and 9.5% in the control (placebo) group (31). Therefore, the relationship between the occurrence of NEC in neonates with HDN and high-dose IVIG treatment has been extensively studied.

In a retrospective study by Figueras-Aloy et al. (32), which included 492 neonates with HDN, it was found that 11 (2.2%) of the neonates who received high-dose IVIG treatment developed NEC, which was much higher than that of 1 (0.3%) in the control group, and the multifactorial analysis showed that high-dose IVIG treatment was an independent risk factor for the development of NEC in neonates with HDN. The study of Hu et al. (33) also supported the above conclusion, and identified the possible reasons being the increase of blood viscosity and the change of vascular tension caused by IVIG input, which can ultimately cause mesenteric ischemia, dilatation of intestinal tubes, intestinal axial membrane necrosis, and intestinal bacterial overgrowth and displacement, leading to the occurrence of NEC. However, there are also meta-analyses (34) and clinical studies (35) claiming that no significant adverse effects have been found with IVIG. These studies indicate that although the role of high-dose IVIg in the occurrence and development of NEC in neonates with HDN still needs further exploration, for the use of high-dose IVIG, clinical indications, infusion speed, concentration, and post-infusion reactions should be strictly monitored in order to avoid adverse consequences such as NEC.

#### 2.2.3 Blood transfusion and double-volume exchange transfusion

Neonates with HDN who are severely anemic often require blood transfusion. Simple transfusion (ST) can be used to correct severe anemia, whereas double-volume exchange transfusion (DVET) serves as a critical intervention for managing severe hyperbilirubinemia refractory to phototherapy and/or eliminating circulating maternal antibodies and bilirubin (36). The mechanisms linking transfusion to intestinal injury are particularly relevant in DVET, as it involves rapid exchange of a large blood volume within a short period.

Nitric oxide (NO) is the main substance that dilates the intestinal blood vessels in newborns, and has the function of relaxing the smooth muscle of the gastrointestinal tract, regulating the blood flow, protecting the integrity, and maintaining the barrier function of the intestinal mucosa (37). The NO activity of erythrocytes in stock blood is significantly decreased, and the blood transfusion in the patient with decreased activity of NO will lead to intestinal vasoconstriction, which further leads to hypoxia, ischemia and even necrosis of the intestinal mucosa (38). In addition, blood viscosity will increase during transfusion, and the neonate's intestinal function is not well developed, which is very sensitive to the change of intestinal hemodynamics, and the neonate is likely to have localized ischemic injury of the intestinal tract (8), which is also a possible reason for the occurrence of NEC. These effects are likely amplified during DVET.

Basic and clinical research on transfusion-associated intestinal injury has provided insights into understanding its risks. Mohankumar et al. (39) used a novel mouse model of transfusion-related NEC and found that the anemic intestine was infiltrated by inflammatory macrophages and developed intestinal injury through the mechanism mediated by Toll-like receptor-4-due to erythrocyte transfusion, and that intestinal injury worsened with the more severity and longer duration of pre-transfusion anemia. The retrospective cohort study by Derienzo et al. (40) indicated that the risk of transfusion-related NEC was negatively correlated with the hematocrit before transfusion [OR = 0.87 (95% CI: 0.79–0.95)]. A study by Maheshwari et al. (41) also showed that neonates had a higher probability of developing NEC within 24–48 h of transfusion, and the more severe the anemia, the greater the risk of developing necrotizing NEC.

Whether to withhold enteral feeding during blood transfusion remains a clinically relevant yet controversial issue. Some studies suggest fasting during transfusion may reduce NEC incidence (42), while others report no significant difference (43, 44). However, the sole systematic review addressing this question (including 7 non-randomized studies, *n* = 7,492) demonstrated that implementing a peritransfusion feeding hold policy during packed red blood cell (PRBC) transfusions in preterm infants significantly reduced the risk of transfusion-associated NEC (TANEC, ≥Bell stage II within 48–72 h post-transfusion) with a relative risk of 0.47 (95% CI: 0.28–0.80) (Evidence quality: moderate) (45). Notably, this protective effect was primarily observed in preterm infants receiving PRBC transfusions. Currently, no high-quality evidence exists to evaluate the efficacy and safety of feeding suspension strategies in neonates with hemolytic disease of the newborn (HDN), particularly those undergoing high-risk double-volume exchange transfusion (DVET). DVET involves substantially larger blood exchange volumes, more pronounced hemodynamic fluctuations, and unique risk factors like severe hemolysis and hyperbilirubinemia—creating a distinct clinical context from standard PRBC transfusions. Therefore, direct extrapolation of findings from preterm PRBC transfusion studies to HDN/DVET cases requires extreme caution.

In summary, clinically distinguishing between “transfusion-associated NEC” (TANEC) and “HDN-associated NEC” (potentially caused by the underlying disease or treatments like IVIG) remains challenging. Nevertheless, evidence strongly suggests that transfusion procedures—especially high-volume DVET—serve as significant contributing factors to NEC development in HDN patients.

### 2.3 Others

It has also been shown that the factors such as immune disorders (46), and low potassium (12) may be associated with the development of NEC in neonates with HDN.

## 3 Conclusion

Neonates with hemolytic disease of the newborn (HDN) face significantly increased risk of necrotizing enterocolitis (NEC), primarily associated with severe anemia, hypercoagulability, intensive phototherapy, intravenous immunoglobulin (IVIG) therapy, blood transfusions, and double-volume exchange transfusion (DVET). As a life-threatening condition, early identification and intervention targeting these risk factors are crucial for improving outcomes.

In clinical practice, risk-based individualized management is recommended:

(1) Judicious assessment of IVIG indications with preference for low-concentration slow infusion.
(2) Prompt correction of severe anemia to alleviate intestinal hypoperfusion.
(3) Strict adherence to defined DVET indications, using fresh blood products, followed by early post-procedural feeding assessment and reintroduction.
(4) Close monitoring of coagulation status and vigilant management of fluid/electrolyte and gastrointestinal tract during phototherapy.

Key practices to avoid:

(1) Indiscriminate IVIG use in mild HDN cases.
(2) Unnecessary delays in indicated transfusions or exchange transfusions due to excessive caution.
(3) Utilization of long-stored blood for large-volume exchange procedures.
(4) Prolonged post-DVET fasting without clinical justification and neglecting feeding tolerance and circulatory assessment during phototherapy.

While current evidence on HDN-associated NEC remains limited, the existing mechanistic understanding and observational data have established a clinically valuable management framework. Future multicenter, large-scale prospective studies are needed to develop more systematic prevention strategies.
